# “Endothelial Antibody Factory” at the Blood Brain Barrier: Novel Approach to Therapy of Neurodegenerative Diseases

**DOI:** 10.3390/pharmaceutics14071418

**Published:** 2022-07-06

**Authors:** Reynald Thinard, Attila E. Farkas, Marta Halasa, Melanie Chevalier, Klaudia Brodaczewska, Aleksandra Majewska, Robert Zdanowski, Maria Paprocka, Joanna Rossowska, Lam Tri Duc, Ruth Greferath, Istvan Krizbai, Fred Van Leuven, Claudine Kieda, Claude Nicolau

**Affiliations:** 1 ALSaTECH, Inc., 300 Market Street, Boston, MA 02135, USA; reynald@alsatech.org (R.T.); melanie.chevalier@alsatech.health (M.C.); ruth.greferath@gmail.com (R.G.); 2 Institute of Biophysics, Biological Research Centre, Eötvös Lorand Research Network (ELKH), 6726 Szeged, Hungary; farkas.attilae@brc.hu (A.E.F.); lamtriduc@brc.hu (L.T.D.); krizbai.istvan@brc.hu (I.K.); 3 Laboratory of Molecular Oncology and Innovative Therapies, Military Institute of Medicine, 04-141 Warsaw, Poland; martahalasa@umlub.pl (M.H.); kbrodaczewska@wim.mil.pl (K.B.); amajewska1@wim.mil.pl (A.M.); rzdanowski@wim.mil.pl (R.Z.); 4 Department of Biochemistry and Molecular Biology, Medical University of Lublin, 20-079 Lublin, Poland; 5 Postgraduate School of Molecular Medicine, Medical University of Warsaw, 02-091 Warsaw, Poland; 6 Hirszfeld Institute of Immunol & Experimental Therapy, Polish Academy of Sciences, 53-114 Wroclaw, Poland; maria.paprocka@hirszfeld.pl (M.P.); joanna.rossowska@hirszfeld.pl (J.R.); 7 Doctoral School of Biology, University of Szeged, 6726 Szeged, Hungary; 8 Institute of Life Sciences, Vasile Goldiş Western University of Arad, 310025 Arad, Romania; 9 LEGTEGG, Katholieke Universiteit Leuven, 3000 Leuven, Belgium; fred.vanleuven@kuleuven.be; 10 Centre for Molecular Biophysics, UPR 4301 CNRS, 45071 Orleans, France; 11 Friedman School of Nutrition Science and Policy, Tufts University, Boston, MA 02111, USA

**Keywords:** cell therapy, gene therapy, immunotherapy, Alzheimer’s disease, amyotrophic lateral sclerosis, β-amyloid, TDP-43, antibody fragments

## Abstract

The failures of anti-β-amyloid immunotherapies suggested that the very low fraction of injected antibodies reaching the brain parenchyma due to the filtering effect of the BBB may be a reason for the lack of therapeutic effect. However, there is no treatment, as yet, for the amyotrophic lateral sclerosis (ALS) despite substantial evidence existing of the involvement of TDP-43 protein in the evolution of ALS. To circumvent this filtering effect, we have developed a novel approach to facilitate the penetration of antibody fragments (Fabs) into the brain parenchyma. Leveraging the homing properties of endothelial progenitor cells (EPCs), we transfected, ex vivo, such cells with vectors encoding anti-β-amyloid and anti-TDP43 Fabs turning them into an “antibody fragment factory”. When injected these cells integrate into the BBB, where they secrete anti-TDP43 Fabs. The results showed the formation of tight junctions between the injected engineered EPCs and the unlabeled resident endothelial cells. When the EPCs were further modified to express the anti-TDP43 Fab, we could observe integration of these cells into the vasculature and the secretion of Fabs. Results confirm that production and secretion of Fabs at the BBB level leads to their migration to the brain parenchyma where they might exert a therapeutic effect.

## 1. Introduction

The discovery of liposomes has stimulated an intense interest, leading to a variety of important applications. The present Festschrift honoring Professor Gregory Gregoriadis, a major pioneer in the field, covers the two most important domains of liposomes research, namely liposomes as agents for drug delivery and as essential elements of vaccines. The work described in this paper is based on the use of conformation-sensitive antibodies raised against “liposomal” antigens. Our previous studies had indicated that the peptide lipidation pattern, the lipid anchor chain length, and the liposome surface charge all significantly alter peptide conformation [1]. Immunization of both mice and monkeys with a novel liposomal vaccine containing β-sheet aggregated lipopeptide [Pal 1–15] induced polyclonal IgG antibodies which specifically recognized β-sheet multimers over monomers or non-pathological native proteins [1]. This is the type of liposomal antigen that we used in the present work.

Neurodegenerative disorders including Alzheimer’s disease (AD) and Amyotrophic Lateral Sclerosis (ALS) are suspected to be caused by the accumulation of misfolded proteins in the brain [2,3,4]. Monoclonal antibodies (mAbs) have been developed to clear misfolded proteins and their aggregates [5,6,7]. To date, all Phase-III clinical trials have been unsuccessful in hitting major endpoints. Despite the high doses administrated, only a small fraction crosses the blood brain barrier (BBB) (less than 1%) [8,9]. The BBB is an extremely efficient filter, not permitting its crossing by 98% of the therapeutic agents tried to date [10,11]. It is a continuous endothelial layer lining the brain microvessels that has sealed cell-to-cell contacts and is sheathed by neural vascular cells and perivascular astrocytic end-feet [11]. The breakdown of the BBB, observed in a number of neurodegenerative diseases, does not favor crossing by therapeutic antibodies, proteins and peptides for several reasons, clearly described by Sweeney et al. [11]. Successful delivery of therapeutic agents across the BBB requires structurally healthy blood vessels, normal vascularization, adequate blood flow and recruitment of solute carrier-mediated transport or receptor-mediated transcytosis systems [11]. Endothelial progenitor cells (EPCs) have been shown to repair damaged Blood-Spinal cord Barrier with beneficial therapeutic consequences [12].

Several studies have documented the regenerative potential of EPCs, and their capacity to sustain a functional vascular system, which is vital to transporting nutrients, signaling molecules, and cells, to the site of tissue injury [13,14,15]. Research from the last decade suggests that EPC’s regenerative ability may be effective in more than just vascular tissue [16,17]. Indeed, the features of EPCs, such as migration, homing and vasculogenesis indicate their potential for use in transplantation or cell-gene therapy for various diseases [18,19,20].

**Figure 1:**
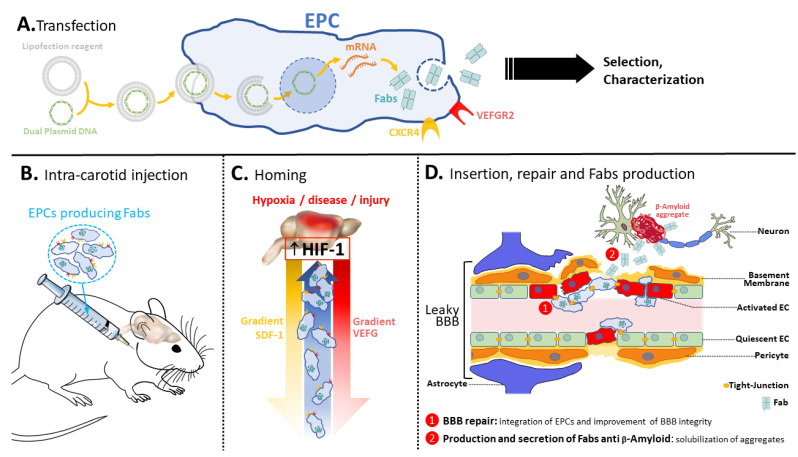
Therapeutic concept. Taking AD as example, the strategy is to combine a cell therapy using the EPCs and the immunotherapy with the secretion of anti-β-amyloid Fabs. After transfection (**A**), the Fab producing EPCs are selected and characterized before being injected into the mice (old or AD mice) (**B**). The transfected EPCs then home to the brain (**C**) where they secrete the solubilizing Fabs (**D**). This system has a dual function: (1) the EPCs themselves, homing to the hypoxic vessels of the pathologic brain and integrating the BBB, repair the damaged BBB occurring in AD, and (2) transfected EPCs secrete, at the BBB, anti-β-amyloid Fabs capable of solubilizing the aggregates.

Herein, we report pre-clinical data as a preliminary proof of concept for the development of a novel approach using ex vivo transfected EPCs as cellular producers of anti-TDP-43 and anti-β-amyloid antibody fragments (Fabs), as depicted on Figure 1, in view of their further use in pathologies involving endothelial damages and BBB leakage. Most neurodegenerative diseases are typified by neuro-inflammation leading to BBB-breakdown. Their homing properties make EPCs, answering to recruitment signals. When injected systemically, EPCs migrate into the BBB in response to local hypoxia. In this case, the cell-mediated gene therapy allows locally secreted Fabs to reach the brain parenchyma, as shown in this paper.

## 2. Materials and Methods

### 2.1. Synthesis of Tetrapalmitoyl-Tris-Lysine-β-Amyloid-Peptide and TDP-43-Peptide

Both β-amyloid 1–16 and TDP-43 311-344 peptides were synthetized by PolyPeptide (Strasbourg, France). For β-amyloid-Peptide, two sequential -palmitoylated lysines were introduced at the peptide’s C terminus. Subsequently, 16 cycles of conventional automated solid-phase peptide synthesis were performed, resulting in the sequence A1–16 appended to the first two palmitoylated lysines. Two further couplings were effected with -palmitoylated lysine. The final deprotection and cleavage from the resin afforded a mixture of lipopeptides which contained the desired tetrapalmitoylpeptide. For TDP-43-Peptide, conventional peptide synthesis was used with no other addition.

### 2.2. Preparation of the Antigens

β-amyloid and TDP-43 liposomal antigens were prepared as previously described [21,22,23]. Liposomes with lipid A and Alum were used as adjuvants to prepare the anti-β-amyloid and anti-TDP-43 vaccines. Then, 1,2-dimyristoyl-sn-glycero-3-phosphocholine (DMPC), 1,2-dimyristoyl-sn-glycero-3-phospho-(1′-rac-glycerol) (DMPG), and cholesterol were mixed in the molar ratios of 0.9:0.1:0.7. Monophosphoryl lipid A, a strong immunomodulator, was added at a concentration of 40 mg per mmol of phospholipids. The peptides were added at a molar ratio peptide to phospholipids of 1:100. Solvents were evaporated, and the resultant film was hydrated with sterile PBS (pH 7.3) to a final phospholipid concentration of 4 mmol and was further homogenized. The liposome suspension was mixed with sterile Alum 15 min before injection (9:1 *v*/*v*).

### 2.3. Immunizations, Antibody Fragments (Fabs) Cloning and Production

The immunizations were conducted by Synaptic Systems (Gottingen, Germany). To raise antibodies against our β-amyloid and TDP-43 antigens, 6–8-week-old females C57Bl6 mice (4 mice antigen-injected, 1 control) were immunized with intra-peritoneal injections in 14-day intervals for 216 and 98 days, respectively, with liposomes presenting the β-amyloid or TDP-43 antigen at their surface. Each injection was 200 µL of 4 mmol antigen suspension containing 8 mM β-amyloid or TDP-43 peptide.

After validation of the specificity of the antibodies produced by ELISA, the mice spleen B-cells were fusioned with P3-X63-Ag8 myeloma line to obtain Hybridomas. Hybridomas were then sequenced by whole-transcriptome shotgun sequencing. After identification of the mature VH and VL regions sequences, they were subcloned in expression vectors to be expressed in Human Embryonic Kidney 293 (HEK293) cells. Cells were transiently transfected with heavy- and light-chain expression vectors and cultures for a further 6 to 14 days. Cultures were harvested and the Fabs were purified using affinity chromatography and analyzed for purity by SDS-PAGE. The amount of Fabs were finally quantified by ELISA.

### 2.4. Solubilization of Anti-β-Amyloid and Anti-TDP-43 Aggregates by Antibodies and Antibody Fragments

Reaction tubes containing 30 µg of β-amyloid 1–42 protein (Bachem, Bubendorf, Switzerland) or 30 µg of TDP-43 protein (LS-Bio, Seattle, Washington, DC, USA) in 10 µL of PBS, pH 7.4 (Gibco), were incubated for 1 week at 37 °C. Aggregation was measured by the thioflavin T (ThT)-binding assay, in which the dye’s fluorescence emission intensity reflects the degree of fibrillar aggregation. Disaggregation was followed after addition of purified antibodies, Fab or supernatants from Fab producing-clones to the preformed fibers (10 µL each). The purified IgG, Fab and an irrelevant control antibody (mouse IgG) were used at a final concentration of 1.5 mg/mL. The irrelevant used as control was a Mouse IgG1 Monoclonal Antibody control (Millipore Sigma, St. Louis, MO, USA), cat #PP100). The same quantity (30 µg) of irrelevant antibody, anti-β-amyloid or anti-TDP-43 antibodies was used. The reaction incubated for 2 days at 37 °C. Fluorescence (excitation: 450 nm; emission: 482 nm) was measured after addition of 1 mL of ThT (3 µM in 50 mM sodium phosphate buffer, pH 6.0) on Fluoromax4C fluorometer (Horiba, Kyoto, Japan).

### 2.5. Plasmids

To create pSF-CAG.InsSP-EGFP, the OG4678 vector (OxGene, Oxford, UK), encoding the CAG promoter, was used as parent vector. PCR was performed to append the human insulin signal peptide to EGFP. The restriction and ligation were performed with the EGFP PCR product and OG4678 to create the final construct for the expression and secretion of EGFP. pl.DualCAG.Hygro.cAb2789 encodes both chains of an anti-βamyloid Fab with optimized peptide signals driven by dual CAG promoters. In addition, a His tag is included to simplify the screening of the Fab production. pl.DualCAG.Hygro.cAb2789 was created with pSF-CAG.InsSP-GFP as the parent vector. The parent vector was restricted and was subsequently ligated with a restricted DNA fragment corresponding to the ubiquitin promoter, a downstream hygromycin resistance marker for cell selection, and a polyadenylation sequence. PCR was performed to append optimized peptide signals to both chains of the anti-β-amyloid Fab encoding sequences (CH1-VH and CL-VL). In addition, a 10-His tag encoding sequence was added to the heavy-chain encoding sequence. Finally, the fragments were subcloned into the parent vector with CAG promoters for both chains to create the final vector expressing and secreting the anti-β-amyloid Fab. Using the same method, pl.DualCAG.Hygro.cAb2508 was synthetized and encodes both chains of an anti TDP-43 Fab. pSF-CAG.InsSP-GFP, pl.DualCAG.Hygro.cAb2789 and pl.DualCAG.Hygro.cAb2508 were verified with restriction digests and Sanger sequencing. All plasmids were commercially prepared with endotoxin levels confirm to be <100 EU/mg (OxGene, Oxford, UK) and diluted to 2 mg/mL in physiological saline.

### 2.6. Cell Line

MAgEC 10.5 cells (murine endothelial progenitor cell line) [Kieda C et al. (2011) Human and murine stem-cell lines: models of endothelial cell precursors/Fasc. European Patent EP 2 524 030 B1] were grown as previously described by Collet et al. [20] in Opti-MEM containing 2% FBS (Gibco, ThermoFisher Scientific, Irvine, CA, USA) at 37 °C with 5% CO_2_.

### 2.7. Cell Transduction with tdTomato Expression Lentiviral Vectors

To establish the MAgEC 10.5 cell line displaying tdTomato expression, the third-generation lentiviral system consisting of pMDLg/pRRE, pRSV-Rev, pMD2.G (a gift from Didier Trono (Addgene plasmid # 12251, 12253, 12259, Watertown, MA, USA)) and expression plasmids pLV [3Exp]-EF1A>{tdTomato}:IRES:Puro (VectorBuilder, Chicago, IL, USA) were used. Lentiviral vectors were produced using Lenti-X™ 293T cell line (Clontech, Takara, Kusatsu, Japan), according to the protocol described by Rossowska et al. [24]. Stable MAgEC 10.5/tdTomato cell line, renamed MagEC 10.5 RT, was obtained after selection with puromycin (10 μg/mL, from Sigma-Aldrich, St. Louis, MO, USA). The transduction efficacy was analyzed for the fluorescence emission of the tdTomato protein in cells by flow cytometry (FACS Aria, Becton Dickinson, Franklin Lakes, NJ, USA).

### 2.8. Cell Transfection and Cloning

MagEC 10.5 RT was seeded at 30,000 cells per well in a 12 wells plate, allowed to adhere for 12 h, and then the medium was exchanged with serum-free Opti-MEM. After 6 h, cells were transfected with Lipofectamine 2000 and Fabs-encoding vectors (pl.DualCAG.Hygro.cAb2789 or pl.DualCAG.Hygro.cAb2508) according to the manufacturer’s instructions. Cells were transfected and kept for further 12 h in a serum-free medium. After 12 h, the hygromycin selection (125 µg/mL) was made. After 24 h of hygromycin selection, the medium was removed and replaced with the fresh one. The medium was changed every 2 days in 2 weeks. After two weeks, the hygromycin-resistant colonies were observed. To obtain proper clones, the cells were detached and seeded at 96 wells plate (1 cell/per well). Selected clones were checked for Fab secretion using western blotting technique.

### 2.9. Western Blot His-Tagged Fab

MAgEC 10.5 clones were cultured for 3–5 days Using OPTI MEM with 2% FBS and 125 ug/mL hygromycin. When the cells reached 90–100% confluence, they were detached by Accutase and counted. Cells were centrifuged, washed with PBS and RIPA buffer with protease inhibitors added. Cells lysates were stored at −20 °C. Then, the lysates were centrifuged at 4000× *g* for 10 min at 4 °C. Protein concentration was quantified using a Pierce^TM^ BCA Protein Assay Kit (ThermoFisher Scientific, Waltham, MA, USA). Cell lysates were solubilized in 4× Laemmli sample buffer and boiled for 5 min at 100 °C. Amounts of 15–20 µg of protein extracts were separated on 10% SDS-PAGE (First: 10 min, 100 V, next: about 60 min, 180 V at 4 °C) and transferred onto the nitrocellulose membrane. Following the transfer, the membrane was blocked with blocking buffer (5% nonfat dried milk in TBS/0.1% Tween-20 (TBST)) for 1 h at RT and incubated overnight at 4 °C with primary antibodies. On the following day, the membrane was washed, then incubated with appropriate horseradish peroxidase-labeled secondary antibodies for 1 h at RT. All the antibodies used in experiment are listed in Table 1. Finally, target proteins were visualized using Western Blotting Luminol Reagent (Santa Cruz Biotechnology, Dallas, TX, USA) according to the manufacturer’s protocol. β-actin was used as a loading control. For MAgECs supernatants, the protocol was the same except for estimating the protein concentration.

### 2.10. Assessment of Surface Markers Using Flow Cytometry

To evaluate expression of surface markers, cells from culture were detached with Acutase Solution (Biolegend, San Diego, CA, USA). The cell suspension was washed with PBS and incubated with TruStain FcX™ PLUS (anti-mouse CD16/32) (#156603 Biolegend)) 10 min on ice to block non-specific binding of immunoglobulin to the Fc receptors. The reagent was used according to the manufacturer’s protocol 0.25 µg per 10^6^ cells in a 100 µL PBS. The cells were then centrifuged, and staining was performed using 10^5^ cells for single marker. Antibodies and isotype controls used in experiment are listed in Table 2; in concentration recommended by the manufacturer in 100 µL Stain Buffer (FBS) (BD Pharmingen™, San Diego, CA, USA). Incubation was continued for 30 min on ice. After this, staining cells were washed twice with 300 µL PBS. Finally, 50 µL of cell suspension in Stain Buffer (FBS) was prepared and cells were analyzed by flow cytometry using CYTOFLEX software v.2.3.0.84 (Becton Dickinson, Franklin Lakes, NJ, USA). The lower threshold was used to exclude debris and live cells with gating (10^4^ cells), according to forward scatter (FSC) × side scatter (SSC) and only singlets were analyzed. Due to the red color of MAgEC RT cells, auto-fluorescence in different channels was checked. APC and Pacific Blue450 were selected as the most useful (the weakest auto-fluorescence). Data were presented as delta MFI to reduce the impact of auto-fluorescence.

### 2.11. Hypoxia Sensitivity of Murine Brain-Derived Endothelial Cells (MBrMECs) Recognition by MAgEC 10.5 RT Cell Line by Adhesion Experiment

The adhesion experiment was performed as previously described [25]. A proportion 1:1 for MAgECs/MBrMECs was used. For hypoxia effect assessment, MBrMECs cells were pre-cultured for 48 h in a cell incubator (19.5% O_2_) or hypoxia chamber (X3 Vivo System, Biospherix, Parish, NY, USA) set for a 1% O_2_ atmosphere for 72 h to reach confluence. After 72 h, MAgEC 10.5 RT cells (control and selected clones) were counted and added in suspension at a ratio of 1:1 to MBrMECs monolayers and were incubated for 20 min at RT with gentle rocking. Un-attached cells were washed off with warm medium. The remaining cells were detached with Accutase and analyzed by flow cytometry. The ratio of MAgEC 10.5 RT to MBrMECs was counted based on the number of events detected in PerCP-positive (MAgECs) to PerCP-negative (MBrMECs) gates.

### 2.12. Endothelial Progenitor Cells Recruitment Assays by BBB Brain Spheroid

The clone selection was performed by video-microscopy based on their ability to be recruited in the BBB. Images were acquired using a Zeiss Axio-Observer.7, fluorescence, and inverted microscope (5× magnification) and analyses were performed with the Zen 2.6 blue edition software (Zeiss, Germany). The time lapse for image taking was 30 min over 72 h. MBrMEC.FVB cells were used to form spheroids as described by Klimkiewicz K et al. [26] and adapted from Choi-Fong Cho et al. [27]. Briefly, a 0.5% methyl cellulose solution in plain Opti-MEM, containing 10^7^ cells/L was deposited as 50 µL drops to allow spheroid formation in hanging drops. Spheroids (20 per well) were further transferred to 24 well plates when the cell amount reached #40,000 cell/spheroid and mixed with a MAgEC-derived cells (5 × 10^5^ cells) suspension in 225 μL methyl cellulose. The plate for cell recruitment was placed under microscopic observation to register the time-lapse-acquired video of the selected area in conditions allowing long term culture (37 °C, for 96 h).

In experiments involving brain organoid-like spheroid formation, performed according to protocol, a composition of cells was used. All cells were stained to identify their localization inside/outside the sphere. MBr MEC.FVB cells were DiO labeled to be detected by λex = 488 nm and λem = 520 nm (GFP channel), pericytes-like (C3H/10T1/2 fibroblasts cultured in Opti-MEM 2% FBS with 1 ng/mL TGF-β during 5–7 days before the experiment) were labeled using the Hoechst stain solution λex = 350 nm and λem = 460 nm (DAPI channel). Astrocytes were DiD labeled to be detected by λex 649 nm and λem = 666 (Cy5 channel). The spheres were cultured in hanging drops (the culture medium was Opti-MEM 2% FBS with 0.25% methylcellulose), and they were analyzed after 2, 3 and 4 days.

### 2.13. Animal Experiments and Immunofluorescence

Subsequently, 8–14-week-old BALB/c mice were anesthetized via inhaled isoflurane 4% (*v*/*v*) in synthetic air for induction and 1–2% (*v*/*v*) for maintenance, using an isoflurane vapourizer (Stoelting, Dublin, Ireland). The depth of anesthesia was monitored by toe-pinch tests. Endothelial precursor cells (EPC)—4 × 10^5^ MAgEC 10.5 RT cells labeled with CellTracker Red (C34552, Thermo Fisher, Waltham, MA, USA) according to the manufacturer’s instructions, MAgEC 10.5 RT cells or MAgEC 10.5 RT anti-TDP-43 cells—were injected into the common carotid artery while the external carotid artery was ligated. 4 h to 7 days later the animals were sacrificed and perfused transcardially with 4 *w*/*w*% formaldehyde. Overnight post-fixation with the same fixative was followed by vibratome sectioning to 30 micrometer thickness. The sections were immunolabeled using antibodies Fab (1:200, A1293 Sigma-Aldrich), PECAM-1 (1:100, NB100-2284, Novus Biologicals, Centennial, CO, USA), claudin 5 (1:200, 35-2500, Invitrogen, ThermoFisher, Waltham, MA, USA), aquaporin-4 (1:100, sc-390488, Santa Cruz Biotechnology, Dallas, TX, USA) and secondary antibodies donkey-α-goat-Alexa+647, donkey-α-rabbit-Alexa+488 donkey-α-mouse-Alexa+488 (1:500, A32849, A32790, A32766, Invitrogen, ThermoFisher, Waltham, MA, USA)). Syto-13 (1:10,000, S7575, ThermoFisher, Invitrogen, Waltham, MA, USA) was used as a nuclear counterstain. Immunofluorescence was recorded using a fluorescence microscope (Axiovert Z1, Zeiss, Budapest Hungary) equipped for super-resolution capable laser scanning confocal microscopy (Stedycon, Abberior Instruments, Göttingen, Germany).

### 2.14. Statistics

The results were analyzed using GraphPad Prism software (version 9.0, GraphPad Software, San Diego, CA, USA). Data are expressed as mean ± standard deviation of the mean (±SD). Statistical differences were considered relevant at *p* < 0.05 (* *p* < 0.05, ** *p* < 0.01, *** *p* < 0.001, **** *p* < 0.0001).

## 3. Results

### 3.1. New Antibodies and Antibody Fragments Directed against β-Amyloid and TDP-43 Proteins

We have raised antibodies against specific sequences of β-amyloid (β-amyloid 1–16) and TDP-43 (TDP-43 311-344), both effectively dissolving in vitro aggregates of the respective proteins. Previous studies with intact antibodies have shown a solubilization of β-amyloid of about 80% [23]. The antibodies, anti-TDP-43 (cAb2508) and anti-β-Amyloid (cAb2789), were then sequenced (Table 3) and the DNA encoding for Fab was inserted in expression vectors prior to producing them via transient expression in Human Embryonic Kidney 293 (HEK293) cells.

**Figure 2:**
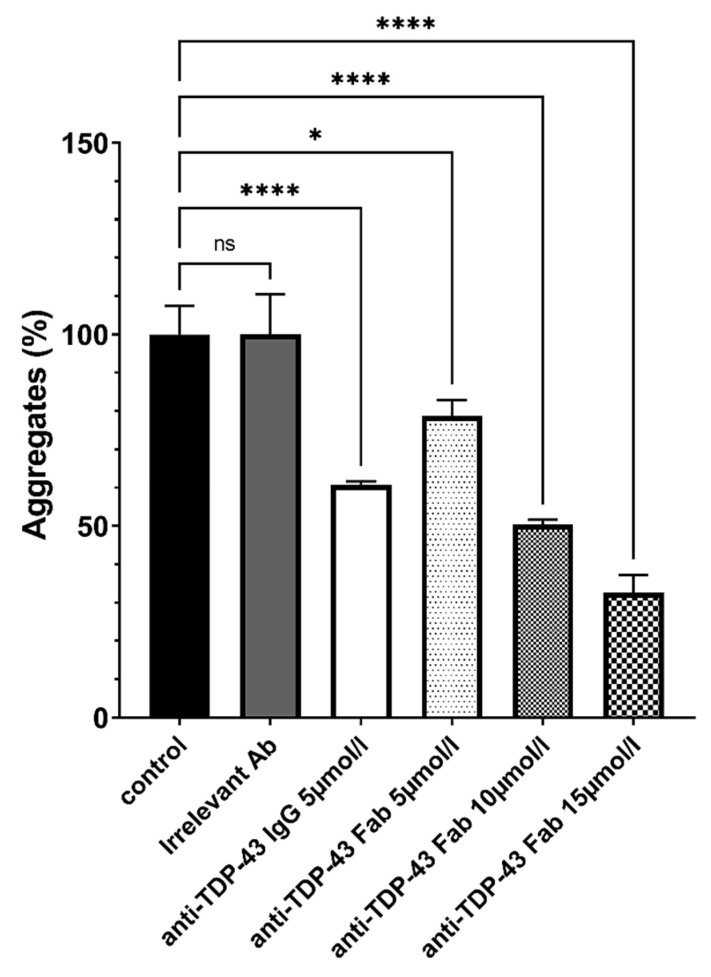
Characterization of the antibodies and Fab produced by clones. The solubilization capacity of the purified antibodies was first tested in vitro on human TDP-43 aggregates using full size anti TDP-43 IgG or anti-TDP-43 Fab at several concentrations. Several concentrations of Fabs were tested to match molar equivalence (5 µmol/L) or mass equivalence (15 µmol/L) compared to anti-TDP-43 IgG (used at 5 µmol/L). The test was also performed with anti-β-amyloid Fab (data not shown). *n* = 3 per group. Statistical differences were considered relevant at *p* < 0.05 (* *p* < 0.05, **** *p* < 0.0001), ns: not significant.

The Fab produced retain the capacity of solubilization of the aggregates in dose-dependent manner (shown for anti-TDP-43 in Figure 2).

### 3.2. Construction of Expression Vectors Encoding Anti-β-Amyloid and TDP-43 Fabs

**Figure 3:**
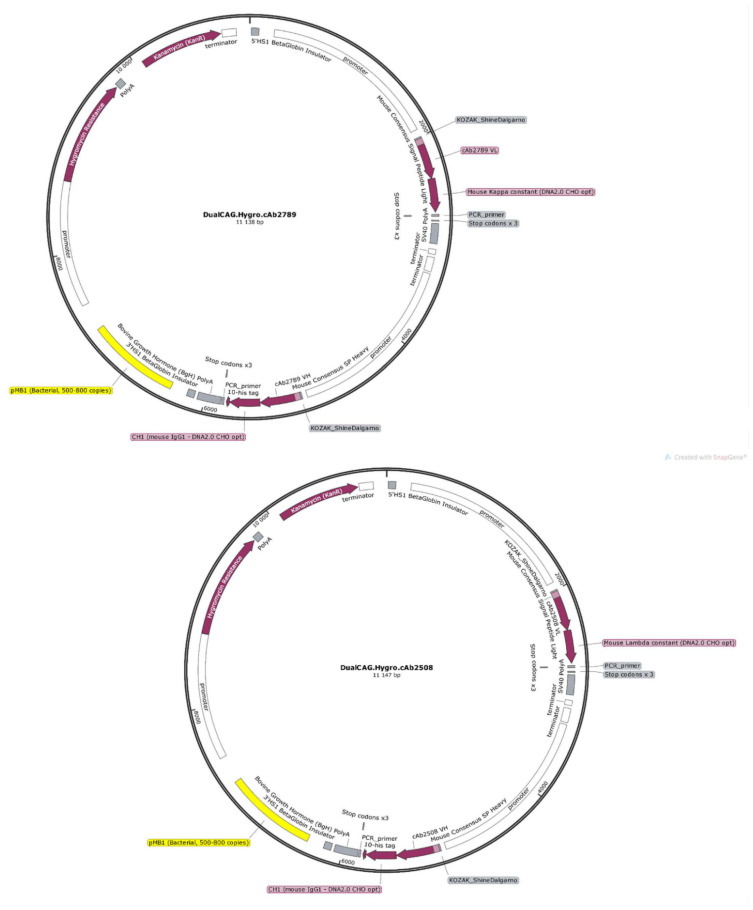
Fabs-encoding vectors maps. To be able to express and secrete anti-TDP-43 and anti-β-amyloid Fabs by endothelial progenitor cells, new expression vectors were created. pl.DualCAG.Hygro.cAb2789 and pl.DualCAG.Hygro.cAb2508 encode the expression of anti-β-amyloid Fab and anti-TDP-43 Fab, respectively. They encode both chains of the Fabs (CH1-VH and CL-VL) with optimized peptide signals driven by dual CAG promoters. In addition, a 10-His tag, a ubiquitin promoter with a downstream hygromycin resistance and a polyadenylation sequence, were added.

The protein sequences of the active Fabs allowed us to build expression vectors containing the respective Fab-encoding sequences, as well as a dual CAG promoter and the specific signal peptides to facilitate the export of the Fab in order to express and produce the Fabs in endothelial progenitor cells (Figure 3) [28].

### 3.3. Preliminary Study with Stable Expression of s-EGFP and Transient Expression of Fab

Prior to transfecting endothelial progenitor MAgEC cells with the anti-TDP-43 and anti-β-amyloid Fab-encoding vectors, we validated transfected endothelial progenitor MAgEC cells for their capacity to produce and secrete exogenous proteins after transfection, using the soluble enhanced Green Fluorescent Protein (s-EGFP) as proof of concept. Different clones were selected by resistance to hygromycin from the transfected MAgEC 10.5 cell line and tested by flow cytometry (Figure S1a,c), in comparison with non-producing clone as control (Figure S1b). The stability of expression and secretion of s-EGFP by the transfected clones were confirmed over several passages (data not shown) and were proportional to the seeded cell number (Figure S1d).

Then, before creating Fab-producing EPCs via stable transfection, the capacity of the endothelial progenitor MAgEC cells to produce Fabs was validated by transient transfections. The MAgEC cells were transfected with anti-β-amyloid Fab-encoding vector by electroporation leading to the production and secretion of Fabs, solubilizing the β-amyloid aggregates, as described by Heller, Thinard et al. [28].

### 3.4. Creation and Characterization of the MAgEC 10.5 RT Anti-β-Amyloid and MAgEC 10.5 RT Anti-TDP-43 Cells: Solubilizing Fab Production and Cell Markers

**Figure 4:**
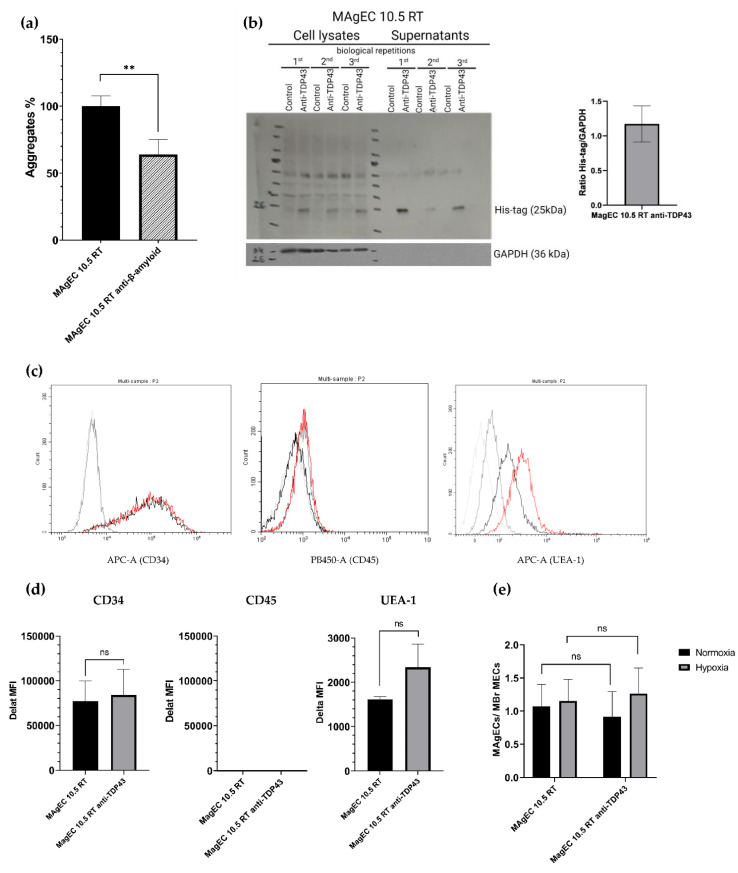
Characterization of the MAgEC 10.5 RT anti-β-amyloid and MAgEC 10.5 RT anti-TDP-43 cells: solubilizing Fab production and cell markers. (**a**), Solubilization capacity of anti-β-amyloid Fabs produced and secreted by the Fab-producing MAgEC 10.5 RT anti-β-amyloid cells. Supernatants from these cells were tested in vitro on human β-amyloid aggregates. *n* = 3 per group. (**b**) Production and secretion of anti-TDP-43 Fab using Western Blot. Fabs are expressed and secreted by the Fab-producing MAgEC 10.5 RT anti-TDP-43 cells. The cell lysates and equal amount of medium from subconfluent cultures after 72 h of culture were used for analysis. The GAPDH was used as control. The densitometry analysis shows the ratio between His-tag and GAPDH in MAgEC 10.5 RT anti-TDP-43 from cell lysates. *n* = 3. (**c**) Flow cytometry analysis of MAgEC 10.5 RT and MAgEC 10.5 RT anti-TDP-43 cells. Grey lines for the three cell markers represent the unstained controls for the MAgEC 10.5 RT (light grey) and MAgEC 10.5 RT anti-TDP-43 cells (dark grey), respectively. Black lines represent expression in MAgEC 10.5 RT cells and red ones the expression on MAgEC 10.5 RT anti-TDP-43 cells surface. Both cell lines are CD34+, CD45- and bind UEA-1. Anti-CD45 labeling was validated using CD45+ cells by flow cytometry (Figure S2). (**d**) Comparative cell-marker expression of MAgEC 10.5 RT and MAgEC 10.5 RT anti-TDP-43 using flow cytometry. The quantitative expression of the markers is expressed in Delta MFI. No difference of expression is observed between the two cell lines, confirming that the Fab producing MAgEC 10.5 RT anti-TDP-43 retains the same phenotype. *n* = 3 per group. (**e**) Hypoxia sensitivity of murine brain-derived endothelial cells (MBrMECs) recognition by MAgEC 10.5 RT by adhesion experiment. The adhesion experiment was performed in proportion 1:1 for MAgECs/MBrMECs in two conditions: hypoxia (grey bars) or normoxia (black bars). After incubation of the MAgEC 10.5 RT or MAgEC 10.5 RT anti-TDP-43 suspension on MBrMECs layer, un-attached cells are removed and then the ratio of MAgEC 10.5 RT to MBrMECs is counted. MAgEC 10.5 RT anti-TDP-43 cells show no significant difference in adhesion capacity compared to the MAgEC 10.5 RT. *n* = 3 per group. Statistical differences were considered relevant at *p* < 0.05 (** *p* < 0.01), ns: not significant.

In order to track the cells in vivo during long duration experiments, the endothelial progenitor MAgEC 10.5 were transduced to express the red fluorescent protein, tdTomato (named MAgEC 10.5 RT) before transfection with the Fab-encoding vectors. We went on to stably transfect these MAgEC 10.5 RT cells with the vectors constructed for expression of anti-β-amyloid and anti-TDP-43 Fabs. The supernatants of selected clones were analyzed in the aggregation assay (shown for anti-β-amyloid in Figure 4a), showing that the supernatants of the anti-β-amyloid MAgEC 10.5 RT cells in culture, after 24 h, were containing Fabs efficiently dissolving β-amyloid aggregates. Indeed, 10 µL of supernatant sample corresponding to 10^4^ cells were able to solubilize 60% of the aggregates as compared to the non-transfected MAgEC 10.5 RT endothelial progenitor cells. As previously shown (Figure 2), the experiments conducted with TDP-43 in vitro had demonstrated the capacity of the expressed anti-TDP-43 Fab to solubilize extensively TDP-43 aggregates. Considering the results obtained with anti-β-amyloid Fabs, both purified and in supernatants of transfected clones, we did not repeat such an experiment with anti-TDP-43 Fabs in cell culture experiments. The production and secretion of Fabs by the clones were also demonstrated by Western Blot (shown for anti-TDP-43 in Figure 4b). We concluded that the cell culture-produced and secreted-Fabs retained their ability to recognize amyloid-β, or TDP-43. All the reported animal experiments were conducted with anti-TDP-43 Fabs expressed by stably transfected MAgEC 10.5 anti-TDP-43. Therefore, we will show the characterization of the MAgEC 10.5 anti-TDP-43 only.

Cytofluorometric analyses of biological markers of the MAgEC 10.5 anti-TDP-43 cells (Figure 4c) demonstrated that the MAgEC 10.5 anti-TDP-43 cells producing and secreting the anti-TDP-43 Fab retained the expression profile of the non-transfected MAgEC 10.5 RT (Figure 4d). Both highly expressed the embryonic marker CD34, and were negative for the immune cells marker CD45, and positive for the glycannic receptors for the characteristic endothelial cell, recognizing Ulex europaeus agglutinin-1 (fucose-specific lectin), showing that the early endothelial progenitor character of the cells was maintained. Moreover, the transfected cells were shown to adhere to the murine brain endothelial cells (MBrMEC), thus confirming their potential to recognize, in vivo, the BBB endothelial cells (Figure 4e).

### 3.5. Endothelial Progenitor Cells Recruitment Assays by BBB Brain Spheroid

**Figure 5:**
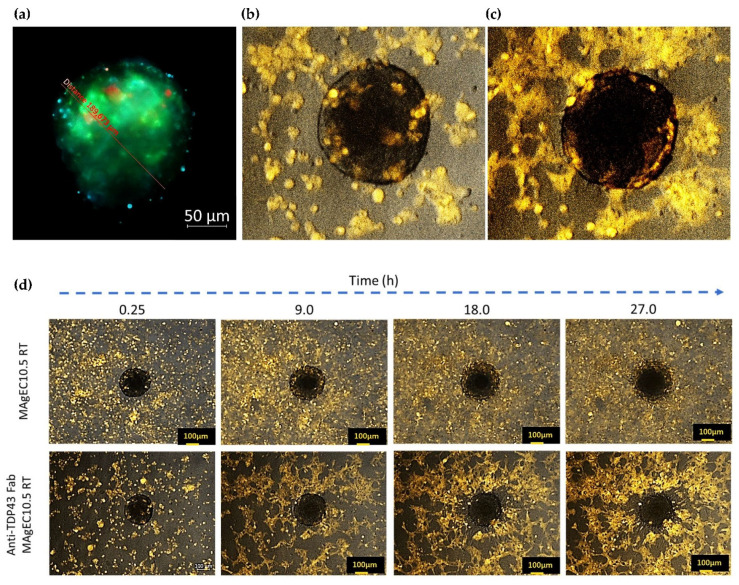
Brain spheroid mediated preferential recruitment of the MAgEC 10.5 RT cells and the Fab-producing MAgEC RT clones. (**a**) Brain spheroid formation and cell identification by fluorescence microscopy. Each cell type is differently labeled: Pericytes-like cells in blue (Hoechst), MBr MEC.FVB in green (DiO), astrocytes in red (DiD). Herein, at day 2. (**b**) Recruitment of the anti-TDP-43 MAgEC 10.5 RT to brain spheroid model. The red fluorescent anti-TDP-43 MAgEC 10.5 RT are detected after 3 h in the spheroids and individually. (**c**) After 30 h the anti-TDP-43 MAgEC 10.5 RT penetrate the brain spheroids, form pseudo vessels and rearrange on the superficial endothelial part of the spheroids. (**d**) Time-related recruitment of the MAgEC10.5 RT cells and the anti-TDP-43 MAgEC RT cells towards the BBB from brain spheroids showing the fast and comparable recruitment of the endothelial progenitors before and after transfection, their pseudo tube formation penetration and selective organization in the brain spheroids.

In order to further validate the use of early endothelial progenitors to carry the therapeutic genes, the in vitro experiments showing their specific and hypoxia sensitive recruitment by the BBB, were performed. For such experiments, brain organoids were composed by three cell types, differently labeled pericytes-like cells (Hoechst labeled), MBr MEC.FVB (DiO labeled), and astrocytes (DiD labeled) and were observed by fluorescence microscopy (Figure 5a). For recruitment experiments the cells entering in the composition of the spheroids were not labeled. The MAgE10.5 RT and the anti-TDP-43 MAgEC10.5 RT clones were visible as they are fluorescent from the Red Tomato protein which they express. Figure 5b,c shows the active recruitment of the cells from the anti-TDP-43 MAgEC10.5 RT by the brain spheroids. Figure 5d shows that the MAgEC 10.5 RT cells are efficiently recruited by the brain spheroids and time-lapse pictures show that, before as after, transfection to produce Fabs, the endothelial progenitor cells are able to penetrate the organoids. Moreover, both cells, before and after transfection, are forming pseudo-tubes. Figure 5b–d also show that the recruited cells are aligning in a BBB-like organization at the external surface of the spheroids as visible on Figure 5c, in accordance with the selective organization of the cells in the spheroid structure as described by Cho, C.F et al. [27].

### 3.6. Homing, Adhesion and Integration of the Injected Cells into the Brain Vasculature

**Figure 6:**
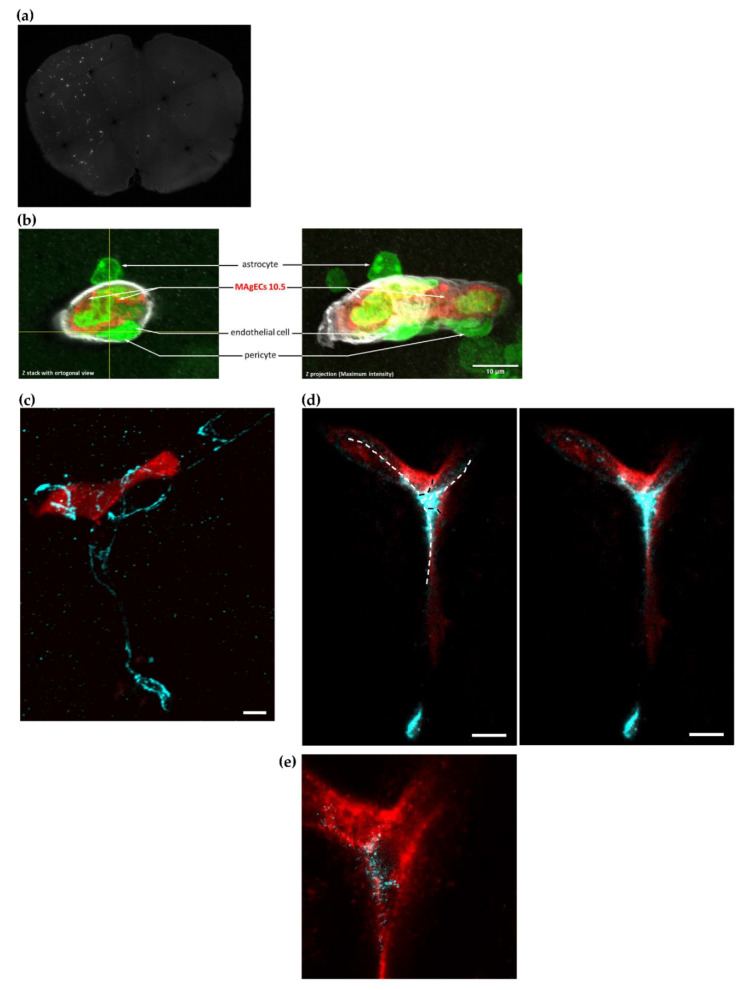
Homing, adhesion and integration of the injected cells into the brain vasculature. (**a**) Detection of the EPCs MAgECs 10.5 labeled by cell tracker red in the brain 4.5 h after intracarotid injection. Detection on sections of 80 µm. The EPCs appear on the sections as white dots. We can see the homing of a fraction of the injected EPCs directly to the brain. See Figure S3 for negative control. (**b**) Microscopic detection of the MAgEC 10.5 cells labeled by cell tracker red. Syto 13 (green), which stains all nuclei, permits the approximate identification of the cells by the position and shape of nuclei. One can identify the astrocytes, the pericytes and the endothelial cells of the vessel wall and the MAgECs inside the vessels. The labeling of collagen IV shows the extracellular matrix and was assessed to identify the relative position of the MAgECs inside the vessels and detect their localisation in the brain microvasculature. (**c**) At 28 h after ICA injection of MAgEC 10.5 (Cell tacker red labeled, red), there are EPCs that do not block the brain microvessel lumen completely, hinting at possible integration of the EPC into the vessel wall. The brain capillary is delineated by PECAM-1 immunolabeling (cyan). The image is a maximum intensity projection of a confocal z-stack. (**d**) Transduced MAgEC 10.5 RT (tdTomato, immunolabeled with anti-RFP, red) were observed in the brain vasculature 7 days after the ICA injection, showing integration into the existing vasculature. The top right capillary branch shows a lumen that appears to be lined with an endothelial cell differentiated from the transduced EPC. The bottom branch appears to contain only partially the red fluorescent cells. Claudin 5 immunolabeling (cyan) shows tight junctions between two edges of the same EPC-derived endothelial cell in both upper branches and between an EPC-derived endothelial cell (red) and another non-labeled endothelial cell (white dashed lines). Between adjacent endothelial cells the tight junction runs the circumference of the vessel, sealing the junction of the two cells (black dashed lines); the right panel shows the image without overlay. The image is a maximum intensity projection of a confocal z-stack. (**e**) STED image from a single optical section of the stack shown in (**d**) reveals the tight junction strand structure at higher resolution (40 nm pixel size). Bars represent 5 µm. The scale bars are 10 µm.

MAgEC 10.5 immortalized endothelial precursor cells fluorescently labeled using Cell Tracker Red were injected into the common carotid artery of adult wild type BALB/c mice. Their brains were sectioned at multiple time points after injection. We could detect in vivo MAgEC 10.5 cells localizing in the brain, as soon as 4 h after injection (Figure 6a). Identification of the astrocytes, the pericytes, the vessel-wall endothelial cells and extracellular matrix collagen IV, permitted to localize the endothelial progenitors MAgEC 10.5 inside the vessels together with their insertion in the BBB. (Figure 6b). Immunolabeling for PECAM-1 and laser-scanning fluorescent microscopy was used to outline brain capillary microvessels around the fluorescent EPCs. Following shortly after injections, EPCs imaged in brain sections appear to fill the capillary lumen. As early as 28 h after injection, EPCs were observed to become located flattening against and adhering to the vessel walls (Figure 6c). Next, for longer duration experiments, MAgEC 10.5 were transduced to express the red fluorescent protein, tdTomato (named MAgEC 10.5 RT), and similarly injected into BALB/c mice. Cells were observed 7 days post-injection for staining for the tight junction protein marker claudin 5. These results showed the formation of tight junctions between the injected MAgEC 10.5 RT as well with unlabeled resident endothelial cells (Figure 6d,e). We concluded that the injected MAgEC 10.5 RT were integrating into the capillary bed.

### 3.7. Exogenic TDP-43 Antibody Secreted by Therapeutic MAgEC Cells Observed in the Brain Vasculature and Brain Parenchyma

**Figure 7:**
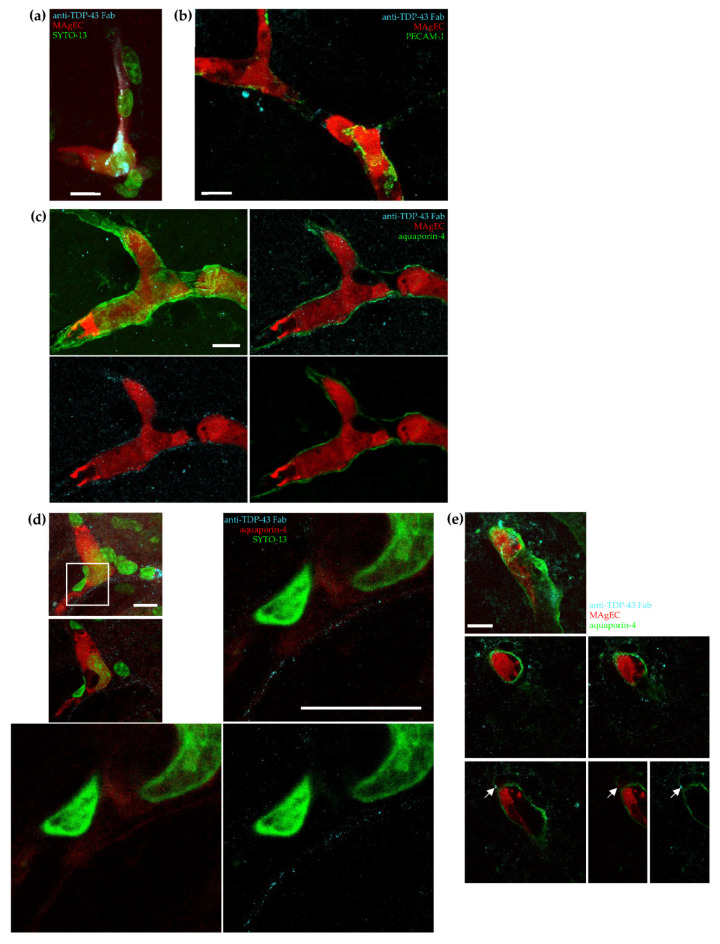
Exogenic TDP-43 antibody secreted by therapeutic MAgEC cells observed in the brain vasculature and brain parenchyma using confocal and super-resolution microscopy. (**a**) MAgEC 10.5 RT anti-TDP-43 cells expressing the fluorescent protein tdTomato (red) and anti TDP-43 Fab fragment (cyan, white in colocalization with red) are discoverable in mouse brain tissue 7 days after intracarotid injection. Nuclear staining (syto-13, green) shows the nuclei of mural cells of a brain microvessel containing MAgEC cells and that of the MAgEC cells themselves. Maximum intensity projection of confocal images. (**b**) Anti-TDP-43 Fab fragments (cyan) produced by MAgEC 10.5 RT anti-TDP-43 cells (red) can pass the blood-brain barrier and localize outside of brain microvessels (labeled using anti-PECAM-1 (green)). The image was taken 48 h after intracarotid injection. Maximum intensity projection of confocal images. (**c**) To further prove that the Fab can penetrate into the brain parenchyma, astrocytic endfeet ensheathing the microvasculature were specifically labeled using antibodies against aquaporin-4 (green). In most brain microvessels observed, the Fab fragments (cyan) secreted by MAgEC 10.5 RT anti-TDP-43 cells (red) localize along the walls of brain microvessels and are spatially indistinguishable from aquaporin-4 labeled astrocytic endfeet surrounding the microvessels on confocal images. The first panel shows maximum intensity projection, subsequent panels present a single optical section showing all channels, MAgEC cells and TDP-43 staining, MAgEC cells and aquaporin-4 staining, respectively. (**d**) The first two smaller panels present confocal maximum intensity projection and single optical section of a brain microvessel containing MAgEC 10.5 RT anti-TDP-43 cells. In this case aquaporin-4 was stained red to allow simultaneous STED imaging with the TDP-43 Fab (cyan). The larger panels show STED images of the smaller area marked with a white square. On most images, it is hard to conclude whether the Fab signal is on the luminal side or the abluminal side of astrocytic end-feet even using super-resolution microscopy. See Figure S4 for negative control. (**e**) However, in some cases Fab signal (arrowheads) is clearly localized in the brain parenchyma outside microvessels containing MAgEC 10.5 RT anti-TDP-43 cells. The parenchymal TDP-43 staining, past the aquaporin-4 signal can coincide with red signal from tdTomato originating from MAgEC cells (arrow). The image shows a maximum intensity projection in the first panel, single optical sections in the following panels, and the last two panels show aquaporin (green) or TDP-43 (cyan) beside the MAgEC cells. The scale bars are 10 µm.

We went on to test our hypothesis that EPCs can be used as vectors for treating neurological disorders by integrating them into the vasculature and secreting therapeutic molecules. Therefore, MAgEC 10.5 RT cells were further modified to also express the anti-TDP-43 Fab (cells named MAgEC 10.5 RT anti-TDP-43). Following characterization of the cells and carotid injection into BALB/c mice, immunolabeling was used to localize the MAgEC 10.5 RT anti-TDP-43 cells and the anti-TDP-43 Fab expressed in relation to the brain microvasculature. The anti-TDP-43 Fab was detected in EPCs located in the brain vasculature 7 days after injection (Figure 7a). Furthermore, anti-TDP-43 Fabs were also observed outside PECAM-1 labeled microvessels that contained red fluorescent EPCs expressing this Fab (Figure 7b).

To test whether the Fab localized into the perivascular space and penetrated into the brain parenchyma, we immunolabeled astrocytic endfeet that ensheath the micro-vasculature by its specific marker aquaporin-4. We observed a distinct localization of the Fabs along the aquaporin-4 stained end-feet (Figure 7c). From these images, it was not possible to differentiate luminal and the abluminal sides of astrocytic endfeet even using super-resolution microscopy (Figure 7d). Nevertheless, we observed sites where the Fabs were clearly localized in the brain parenchyma, past the aquaporin-4 signal. It is noteworthy that the extravascular anti-TDP-43 Fab appeared co-located with tdTomato originating from MAgEC 10.5 RT anti-TDP-43 cells, suggesting vesicular localization (Figure 7e).

## 4. Discussion

The approach to a therapy for neurodegenerative diseases described in this work adds to the already existing therapeutical uses of EPC [20,28,29] by using transfected EPCs that express active antibody fragments or other therapeutic proteins [20]. This system has a dual function: (1) the EPCs themselves repair the damaged BBB occurring in both ALS and AD–, and (2) transfected EPCs secrete, at the BBB, the anti-TDP-43 and anti-β-amyloid Fabs capable of solubilizing the aggregates of TDP-43 and β-amyloid. The results presented show that it is possible to establish a production of antibody fragments by transfected EPCs, and that these Fabs bind to aggregates of TDP-43 and β-amyloid, solubilizing a significant fraction of them in a concentration-dependent manner. The in vivo conditions were not expected to bring a treatment, but the proof-of-concept, validating the presently designed cell-gene therapy. It shows the effective presence of the EPCs in the BBB and their further incorporation among the brain endothelium. Moreover, the transfected EPCs 7-days post-injection became stained for the marker claudin 5, indicating the formation of tight junctions between the injected MAgEC 10.5 RT and unlabeled resident endothelial cells. Thus, it clearly appears that the injected cells were integrated in the capillary bed.

The experimental results further showed localization of the Fabs in the perivascular space and in the brain parenchyma. Immunolabeling of the astrocytes end-feet with its specific marker aquaporin-4 showed a clear localization of the Fabs in the brain parenchyma, past the aquaporin signal. The results obtained demonstrate that antibodies produced at the BBB by EPCs transfected with our expression vectors are capable of penetrating the brain parenchyma. The results suggest that the EPCs could be transfected not only with vectors expressing antibody fragments, but with vectors expressing other exogeneous proteins that are normally blocked by the BBB. The in vivo application described in this paper brings the validation of the concept documented, in turn, by the in vitro experiments. The latter show that it is possible to observe a preferential recognition of the BBB cells by EPC derived from early endothelial progenitors. Those cells were previously described as possible tools in response to brain-disease signals [30] and a model for endothelial cell recruitment in all hypoxia-dependent diseases which range from cardiovascular to cancerous pathologies [20]. In all such diseases, the early endothelial progenitor cells recruitment is a repair mechanism naturally occurring, but often insufficiently efficient. The origin of the endothelial progenitor is still a matter of discussion regarding their localization, in the bone marrow or the stem niches in the tissue vicinity. In both cases, the natural role and purpose of their recruitment is to restore a proper vascularization, allowing for an efficient blood flow and alleviating the pathologic hypoxia, which is the cause that maintains the condition for the progression of the disease [31].

The in vitro assays performed indicate that a better recognition of the organo-specific brain-derived endothelial cells are recognized more efficiently when they are in hypoxia-treated, while hypoxia did not affect the endothelial progenitor cells adhesion properties. This was confirmed by the active recruitment of the early EPCs by a pseudo-organ mimicking the blood-brain barrier and which actively attracted the EPCs and caused rearrangements into pseudo-tube formation. The models used indicated that such transfected EPCs kept their proangiogenic properties, confirming that cells with the corresponding phenotype are good candidates for future repair strategies, as well as for gene-expression delivery. Indeed, the successive transfection steps, although they necessarily result in modifying the cells to some extent, also allowed us to select clones that have kept the early endothelial progenitor cells properties and phenotypic characteristics, as well as the ability to synthesize and secrete the desired anti TDP-43- or anti-β-amyloid Fab. The first in vivo experiments presented herein were performed with a view to obtaining a basic proof-of-concept, verifying that the main requirements for a valid in vivo development of the cell-gene therapy are fulfilled for neurodegenerative-diseases treatment. The above-described in vivo data showing that the blood-borne EPCs were able to incorporate into the BBB and express the therapeutic protein validate the approach and permit future expansion of the strategy by application of the treatment to ALS-diseased mice. It is expected that such mice, as the SOD1-mutated line, might offer the required conditions expressing a hypoxic BBB and the production of TDP-43 in the brain parenchyma, which will permit the long-term measurement of the recovery of the movement capacity in the treated animals [32].

Concerning the clinical development of this approach, we realize that using an autologous approach in a clinical context is tedious and hampered by the time required by the engineering of the EPCs, from the harvesting of the patient’s EPCs up to the reinjection as transfected EPCs.

Taking into consideration this limit and based on the proof-of-concept showed in this article, a cell-engineering project, starting from pluripotent stem cells and resulting in an unlimited resource for EPC production with constitutive expression of therapeutic antibody fragments is under consideration in our laboratory. Once achieved, we will measure the half-life of the injected cells, confirm the absence of immunogenicity of the cells, determine the percentage of the cells homing to the target site, and more.

## 5. Conclusions

In summary, ex vivo transfection of endothelial progenitor cells with expression vectors led to the production of antibody fragments (Fabs) retaining their ability to recognize TDP-43 and β-amyloid aggregates. When such cells were injected into mice, they were detected at the BBB, where they integrated, continuing Fab production. Immunofluorescence studies showed that some of the injected cells form tight junctions among them and with the unlabeled resident endothelial cells. The natural repair process of vessels in pathologic angiogenesis-induced damage is the recruitment of EPCs from the bone marrow or mobilization upon paracrine signaling of endothelial progenitors from vicinal tissues [20,33,34]. Combining the BBB-repairing by the EPCs with the production in situ of antibodies or Fabs directed against TDP-43 or β-amyloid might lead to a cell-based gene and immunotherapy of neurodegenerative and many other diseases.

## Figures and Tables

**Table 1:** List of antibodies used for Western Blot.

Primary Antibodies	Catalog Number
Anti-Histag Chimeric	Merck, SAB5600096
Anti-Mouse IgG (Fab specific)	ThermoFisher, 31413
**Secondary antibodies**	
Anti-Histag Chimeric secondary	Merck, A1293
Rabbit anti-Goat IgG (H + L) secondary	ThermoFisher, 81-1620

**Table 2:** List of antibodies used for flow cytometry.

Antibody	Catalog Number	Details
CD34	152207 (Biolegend)	Rat IgG2a Brilliant Violet 421™
CD45	103111(Biolegend)	Rat IgG2b APC
UEA-1	DL-1069 (Vector Labratories)	DyLight649 labeling
Isotype Control Antibody	400511 (Biolegend)	Rat IgG2a APC
Isotype Control Antibody	400611(Biolegend)	Rat IgG2b APC

**Table 3:** Protein sequences of the anti-TDP-43 and anti-β-amyloid antibodies. The table presents the protein sequences of the different domains of the anti-TDP-43 and anti-β-amyloid antibodies: The signal peptides (sig. pep.) for every chain, the heavy chain with its variable domain (VH) and constant domain, the light-chain sequence with its light chain variable domain (VL) and constant domain. The type of heavy-chain constant region (HC type) and light-chain constant region (LC type) are also indicated.

Clone Name	**Primary Heavy Chain Protein Sequence**			
	HC Type	Sig. Pep.	VH	Constant
**anti-TDP-43 antibody (cAb2508)**	Mouse IgG2b	MEWIWIFLFILSGTAGVQS	QVQLQQSGAELARPGASVKLSCKASGYTFTSYGISWVRQRTGQGLEWIGEIYPRRGNTYYNEKFKGKATLTAYKSSGTAYMELRSLTSEDSAVFFCARGGIYYGNLFDYWGQGTTLTVSS	AKTTPPSVYPLAPGCGDTTGSSVTLGCLVKGYFPESVTVTWNSGSLSSSVHTFPALLQSGLYTMSSSVTVPSSTWPSQTVTCSVAHPASSTTVDKKLEPSGPISTINPCPPCKECHKCPAPNLEGGPSVFIFPPNIKDVLMISLTPKVTCVVVDVSEDDPDVRISWFVNNVEVHTAQTQTHREDYNSTIRVVSALPIQHQDWMSGKEFKCKVNNKDLPSPIERTISKIKGLVRAPQVYILPPPAEQLSRKDVSLTCLVVGFNPGDISVEWTSNGHTEENYKDTAPVLDSDGSYFIYSKLDIKTSKWEKTDSFSCNVRHEGLKNYYLKKTISRSPGK
	**Primary light chain protein sequence**			
	**LC type**	**Sig. pep.**	**VL**	**Constant**
	Mouse lambda	MAWISLILSLLALSSGAIS	QAVVTQESALTTSPGETVTLTCRSSTGAVTTSNYANWVQEKPDHLFTGLIGGTNNRAPGVPARFSGSLIGDKAALTITGAQTEDEAIYFCALWFSNHWVFGGGTKLTVLG	QPKSSPSVTLFPPSSEELETNKATLVCTITDFYPGVVTVDWKVDGTPVTQGMETTQPSKQSNNKYMASSYLTLTARAWERHSSYSCQVTHEGHTVEKSLSRADCS
	**Primary heavy chain protein sequence**			
	**HC type**	**Sig. pep.**	**VH**	**Constant**
**anti-β-Amyloid antibody (cAb2789)**	Mouse IgM	MEWPLIFLFLLSGTAGVQS	QVQLQQSGAELVKPGASVKISCKASGYAFSNYWMNWVKQRPGKGLEWIGQIYPGDGDTNYNGKFKGKATLTADKSSSTAYMQLSSLTSEDSAVYFCARGDYWGQGTTLTVSS	ESQSFPNVFPLVSCESPLSDKNLVAMGCLARDFLPSTISFTWNYQNNTEVIQGIRTFPTLRTGGKYLATSQVLLSPKSILEGSDEYLVCKIHYGGKNKDLHVPIPAVAEMNPNVNVFVPPRDGFSGPAPRKSKLICEATNFTPKPITVSWLKDGKLVESGFTTDPVTIENKGSTPQTYKVISTLTISEIDWLNLNVYTCRVDHRGLTFLKNVSSTCAASPSTDILTFTIPPSFADIFLSKSANLTCLVSNLATYETLNISWASQSGEPLETKIKIMESHPNGTFSAKGVASVCVEDWNNRKEFVCTVTHRDLPSPQKKFISKPNEVHKHPPAVYLLPPAREQLNLRESATVTCLVKGFSPADISVQWLQRGQLLPQEKYVTSAPMPEPGAPGFYFTHSILTVTEEEWNSGETYTCVVGHEALPHLVTERTVDKSTGKPTLYNVSLIMSDTGGTCY
	**Primary light chain protein sequence**			
	**LC type**	**Sig. pep.**	**VL**	**Constant**
	Mouse kappa	MESQTQVLMFLLLWVSGACA	DIVMTQSPSSLAMSVGQKVTMSCKSSQSLLNSSNQKNYLAWYQQKPGQSPKLLVYFASTRESGVPDRFIGSGSGTDFTLTISSVQAEDLADYFCQQHYNTPLTFGAGTKLELK	RADAAPTVSIFPPSSEQLTSGGASVVCFLNNFYPKDINVKWKIDGSERQNGVLNSWTDQDSKDSTYSMSSTLTLTKDEYERHNSYTCEATHKTSTSPIVKSFNRNEC

## Data Availability

Not applicable.

## Supplementary Materials

The following supporting information can be downloaded at: https://www.mdpi.com/article/10.3390/pharmaceutics14071418/s1. Figure S1. Flow cytometry analysis of the expression of s-EGFP by the transfected MAgEC 10.5 s-EGFP and quantification of the secretion of s-EGFP by MAgEC 10.5 s-EGFP. Figure S2. Validation of anti-CD45 labeling using CD45+ cells by flow cytometry. Comparison to MAgEC 10.5 wt and MAgEC 10.5 anti-TDP-43 which are both CD45−. Figure S3. Negative control for homing, adhesion, and integration of the injected cells into the brain vasculature. Figure S4. Negative control for the secretion of the exogenic TDP-43 antibody by therapeutic MAgEC cells observed in the brain vasculature and brain parenchyma.
