# Supplementary material for: “Endothelial Antibody Factory” at the Blood Brain Barrier: Novel Approach to Therapy of Neurodegenerative Diseases
Source: Pharmaceutics. 2022 Jul 6;14(7):1418. doi: 10.3390/pharmaceutics14071418 (PMC9320725; doi:10.3390/pharmaceutics14071418)
Supplement: Supplementary file 1 [file pharmaceutics-14-01418-s001.zip › pharmaceutics-1763677-supplementary.pdf]

## Supplementary data

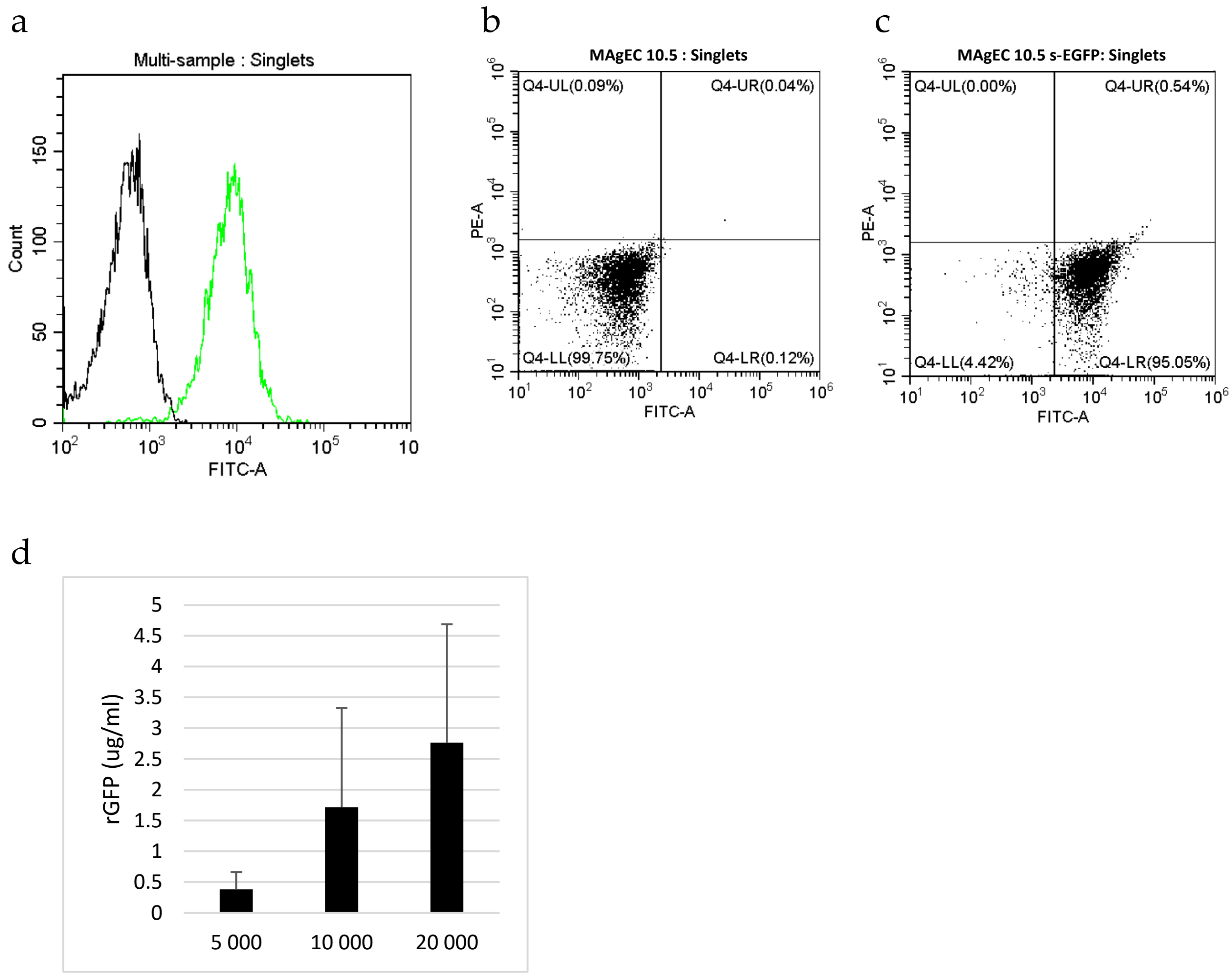


**Figure S1. Flow cytometry analysis of the expression of s-EGFP by the transfected MAgEC 10.5 s-EGFP and quantification of the secretion of s-EGFP by MAgEC 10.5 s-EGFP.** Expression of s-EGFP MAgEC 10.5 s-EGFP (**a**. green line on histogram, dot plot **c**.) in comparison with non-transfected cells MAgEC 10.5 analyzed as control (**a**. grey line on histogram, dot plot **b.**) **d,** Secreted s-EGFP in supernatant was quantified after 24 h using several density MAgEC 10.5 s-EGFP. The concentration of s-EGFP secreted in supernatant is expressed as a function of the initial seeding cell density, calculated basing on rGFP curve. n=3.

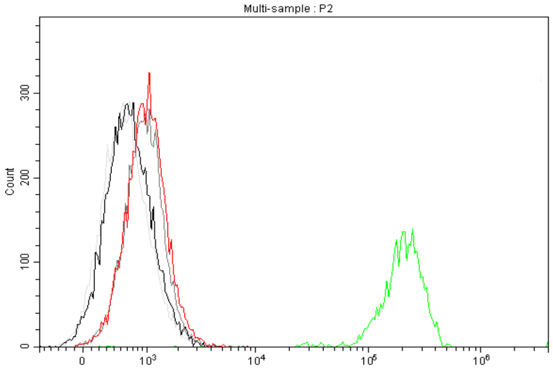


**Figure S2. Validation of anti-CD45 labeling using CD45+ cells by flow cytometry. Comparison to MAgEC 10.5 wt and MAgEC 10.5 anti-TDP-43 which are both CD45-.** Positive control for **Figure 4. c.** Flow cytometry analysis of MAgEC 10.5 RT and MAgEC 10.5 RT anti-TDP-43 cells. Grey lines represent the unstained controls for the MAgEC 10.5 RT (light grey) and MAgEC 10.5 RT anti-TDP-43 cells (dark grey), respectively. Black line represents expression in MAgEC 10.5 RT cells and the red one the expression on MAgEC 10.5 RT anti-TDP-43 cells surface. Mouse whole blood was used as positive control (green line) to validate anti-CD45 labeling and to confirm that the negative phenotype of MAgEC 10.5 RT and MAgEC 10.5 RT anti-TDP-43 cells occurs due only to the non-expression of the marker.

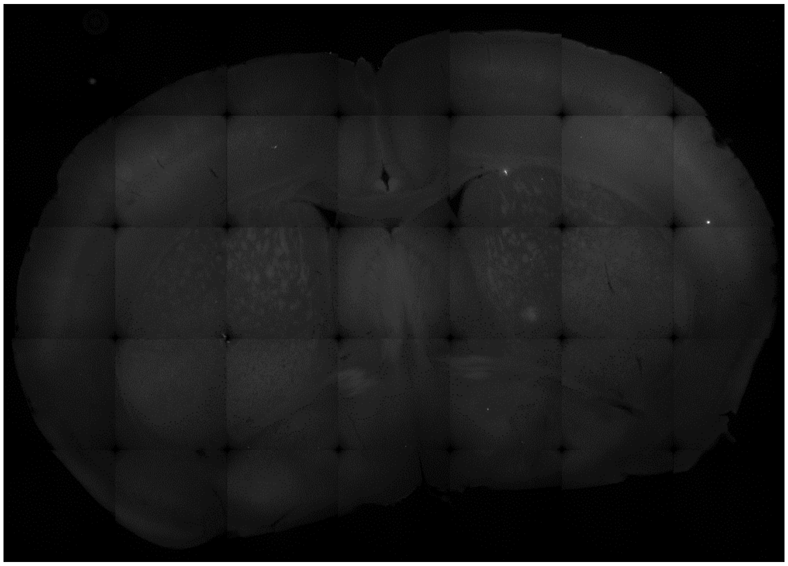

**Figure S3. Negative control for homing, adhesion, and integration of the injected cells into the brain vasculature.** These pictures represent the control for the **Figure 6. a.** showing the detection of the EPCs MAgECs 10.5 labeled by cell tracker red in the brain 4.5 hours after intracarotid injection. In that control, no cells where injected. The same sample preparation and microscope settings were used as for **Figure 6. a.**

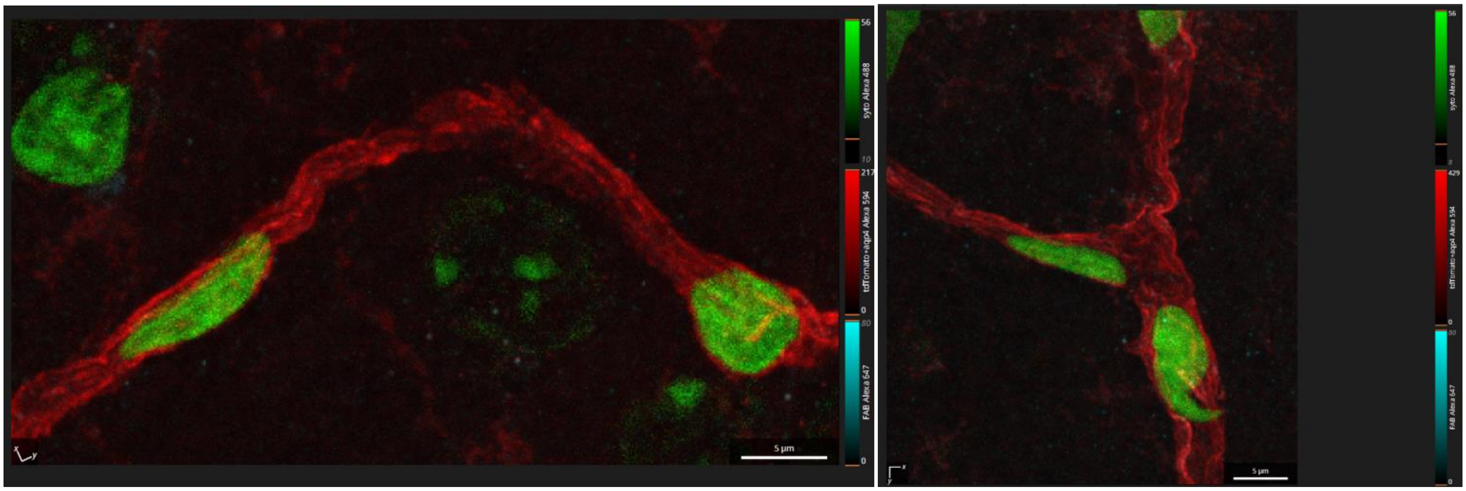


**Figure S4. Negative control for the secretion of the exogenic TDP-43 antibody by therapeutic MAgEC cells observed in the brain vasculature and brain parenchyma.** These pictures represent the control for the **Figure 7. d.** Brain sections were used from mice not injected with Fab secreting MAgEC, but otherwise the samples were handled the identically regarding intracarotid injection, sectioning, and immunostaining. The same microscope settings were used as for **Figure 7. d.**
